# News and Coming Events

**Published:** 1908-12-12

**Authors:** 


### News and Coming Events.

The Poisons and Pharmacy Bill passed the second reading stage in the House of Commons on Thursday, December 3.

At the last meeting of the managers of the Edinburgh Royal Infirmary, Mr. John William Struthers, M.B., F.R.C.S. (Edin.), was appointed assistant surgeon, thus filling the vacancy caused by the promotion of Mr. David Wallace to the post of surgeon.

A dinner was given on Monday, November 23, at the Trocadero Restaurant to Sir Robert W. Burnet, hon. physician to H.R.H. the Prince of Wales, to celebrate his recent knighthood. Sir J. Dickson-Poynder, M.P., presided, and among the medical men present were Sir Thomas Barlow and Inspector-General J. Porter, director-general of the medical department of the Royal Navy.

The Glasgow University Club, London, held its autumn dinner on November 27, under the chairmanship of Professor Archibald Barr, at the Trocadero Restaurant. The guest of the evening was Dr. C. O. Hawthorne, whose honorary secretaryship of the club from 1901 to 1908 has been so successful. Professor Barr proposed "The University and the Club." Dr. Guthrie Rankin proposed "The Health of Dr. Hawthorne," and spoke of his work on behalf of the club. The toast was received with enthusiasm. After Dr. Hawthorne had responded, Sir Donald MacAlister proposed "The Guests," which was replied to by Professor James Little.

The Lord President of the Council has appointed a departmental committee to consider the working of the Midwives Act, and in particular with reference to the supply of midwives and the cost of training, the remuneration of medical men summoned on the advice of midwives under the rules in pursuance of the Act, and the delegation of their powers by county councils under the Act. The chairman of the committee is Mr. Almeric W. Fitzroy, Clerk of the Council, and the members are: Mrs. Charles Hobhouse, Mr. J. S. Davy, C.B., assistant secretary, Local Government Board; Dr. A. H. Downes, medical inspector for Poor-law purposes, Local Government Board; Dr. F. H. Champneys, chairman of the Central Midwives Board; and Mr. John Pedder, of the Home Office. Mr. H. J. Stanley and Mr. F. J. Welch, will act as joint secretaries.

The Hon. Sydney Holland presided on December 2 at the quarterly General Court of the London Hospital. The Committee of Management announced that the amount realised in connection with the quinquennial appeal (cash and promises) was £78,303. Two additional promises of £2,000 each had been received from a lady and gentleman, who wished to remain anonymous, on condition that a further £2,000 was raised in donations before the end of the year for the general hospital work, and also £2,000 for the women's wards. Princess Hatzfeldt had kindly given a complete set of Dr. Tyrnauer's hot-air baths for the treatment of rheumatism, sciatica, gout, etc. Dr. Tyrnauer had himself spent several days in instructing the nurses and explaining to the staff the methods of administering the treatment. In view of the fact that no private interests would be affected, the committee decided to take in paying patients, many applications having already been received. On the motion of the Chairman, Mr. Russell Howard was elected on the surgical staff in the room of the late Mr. Harold Barnard, and the report was adopted.

Dr. Frederick Langmead, M.D.Lond., M.R.C.P.Lond., has been appointed assistant physician to the *Dreadnought* Hospital, Greenwich, and casualty physician to St. Mary's Hospital.

The West of England Eye Infirmary has received £2,460 towards the £3,000 required to erect a new wing which is to be built to celebrate the "centenary" of the institution. It is hoped that the remaining £540 will shortly be subscribed.

The distribution of prizes and an address to the students of Charing Cross Hospital Medical School by John H. Morgan, Esq., C.V.O., M.A., F.R.C.S., consulting surgeon to the hospital, will take place in the out-patients' hall at the hospital on Friday, December 18, at 4 o'clock. Tea and coffee will be served at the conclusion of the proceedings.

The *Dublin Gazette* announces that letters patent bearing date December 2 have passed the Great Seal of Ireland, constituting and founding a new college having its seat in Dublin under the name of University College, Dublin; granting a new charter to Queen's College, Cork, under the name and style of University College, Cork; and a new charter to Queen's College, Galway, under the name and style of University College, Galway.

Dr. F. Walker Mott, F.R.S., has been elected Fullerian Professor of Physiology in the Royal Institution. Among the forthcoming lecture arrangements at the Royal Institution are the following: Professor Karl Pearson, two lectures on Albinism in Man; Dr. F. Walker Mott, six lectures on the Evolution of the Brain as an Organ of Mind. The Friday evening meetings will begin on January 22, when Dr. Alfred Russel Wallace, O.M., will deliver a discourse on "The World of Life: as Visualised and Interpreted by Darwinism."

The fifty-third annual festival dinner of the Poplar Hospital for Accidents was held on December 7 at the Holborn Restaurant. Mr. J. G. Broodbank, secretary of the London and India Docks Company, presided, and among those present were Captain Lord Herbert Scott, the Hon. Sydney Holland (Chairman of the Hospital), the Hon. Noel Farrer, Count Stomm, Mr. Crooks, M.P., and the Mayors of Paddington and Poplar. The Hon. Sydney Holland said that the hospital had never been in debt. Nearly 1,000 accidents were treated there during every week of last year. During the evening subscriptions to the amount of £3,363 19s. 2d. were announced.

In a discussion in the Senate of the University of Cambridge on December 3 Professor Sir T. Clifford Allbutt opposed the report of the general board, which recommended the abolition of the University lecturer in midwifery. He urged the importance of the subject, and the need for some official representative thereof, in view of the large part it played in the final examinations for the M.D. degree. Dr. Laurence Humphry supported the proposal to discontinue the lectureship, on the grounds that the subject now came later in the medical student's curriculum, and was chiefly studied by Cambridge students at the large hospitals away from the University. Mr. R. T. Wright objected to the form in which the proposal came before the Senate, inasmuch as it afforded no indication of the numbers of the medical board who supported the proposed abolition of the lectureship.

The *Journal of Gas Lighting* announces that, out of the eventual residue of the estate of the late Sir George Livesey (subject to the life interest of Lady Livesey), £10,000 is to be paid to the King's Hospital Fund.

The annual meeting of the Surgical Aid Society was held on December 8 at the Mansion House, under the presidency of the Lord Mayor (Alderman Sir George W. Truscott). Sir H. B. Marshall, Major-General the Hon. Sir F. W. Stopford, the Mayor of Croydon, the Rev. Thomas Phillips, Mr. F. F. Belsey, Mr. A. P. Gould, and Mr. Samuel Watson (Treasurer) were also present. The report referred to the continued growth and prosperity of the society, and stated that the net income of the general fund for the year under review was £24,498, an increase of £1,603 upon that of the previous year. There had been 38,348 appliances supplied during the past twelve months, bringing up the total since the foundation of the society to over 611,000. The number of patients relieved during the year was 24,770, an average of 476 per week.

The Life Assurance Medical Officers' Association on December 2 discussed further the paper read on November 4 by Sir Thomas Oliver, physician to the Royal Victoria Infirmary, Newcastle-upon-Tyne, on "Some Medical and Insurance Problems Arising out of Recent Industrial Legislation." Dr. E. Glover Lyon, president of the association, again occupied the chair, and in opening the proceedings offered Professor Oliver the congratulations of the Association on his recent knighthood. Dr. Mechan, of Glasgow, referred to the effects of the Workmen's Compensation Act on the conditions of labour in the shipbuilding and engineering yards on the Clyde. He said his experience was that the working classes had not become more thrifty, litigation was more often resorted to by workmen who had received slight injuries or none at all, and malingering was on the increase, illness which used to be feigned in order to shirk labour now being feigned in order to obtain money. Sir Thomas Oliver, in his reply, advocated the speedy settlement of compensation claims, but not the payment of lump sums. He pointed out that there had been an increase in the number of fatal accidents in spite of the greater precautions now taken in factories. It was to the credit of the medical profession that its members enjoyed the confidence of both employers and employed.

The National Hospital for the Paralysed and Epileptic, in Queen Square, Bloomsbury, which was incorporated as a memorial to the late Duke of Albany, will next year celebrate its jubilee, having been founded in 1859. The Duchess of Albany, as president of the institution, is asking for £50,000 with which to commemorate its fifty years' work in the treatment of nervous diseases; and considerable progress has already been made. Several Lords-Lieutenant have issued circulars in their counties, and the sums already received include £158 from Middlesex (per the Duke of Bedford), £228 from Northamptonshire (per Lord Spencer), and £355 from Surrey (per the Hon. Henry Cubitt). The Lord Mayor intends to ask provincial and suburban Lord Mayors and Mayors to assist him in promoting the success of the fund. The trustees of the Zunz Fund have promised £5,000 towards an endowment fund. The Duchess of Albany has intimated that if a sufficient sum is raised, and the collection is of a really national character, she will feel justified in requesting the presence of the King (who, in 1885, opened the main building of the hospital) at some ceremonial to be attended by the donors or their representatives. The Archbishop of Canterbury has promised to hold a jubilee thanksgiving service in the hospital chapel.

